# CULTURAL AND POSITIVE ADAPTIVE FACTORS INFLUENCING DIVERSE PARTNER CAREGIVERS THROUGH A RESILIENCE FRAMEWORK

**DOI:** 10.1093/geroni/igae098.1626

**Published:** 2024-12-31

**Authors:** Shandra Burton, Olimpia Paun, Todd Ruppar, lynn Mohr, Chuka Emezue, Sarah Szanton

**Affiliations:** Marian University Indianapolis School of Nursing, Indianapolis, Indiana, United States; Rush University, Chicago, Illinois, United States; Rush University College of Nursing, Chicago, Illinois, United States; Rush University College of Nursing, Chicago, Illinois, United States; Rush University College of Nursing, Chicago, Illinois, United States; Johns Hopkins University, Baltimore, Maryland, United States

## Abstract

Collectively, Alzheimer’s disease and related dementias (ADRD) are the 5th leading cause of death for Americans aged 50 years and older and remains a critical public health issue for partner caregivers (i.e., committed, married, or cohabiting individuals). ADRD partner caregivers often face high levels of stress and burden contributing to their poor physical and psychological health outcomes. Research on positive adaptive factors (PAFs) among caregivers of individuals with Alzheimer’s disease and related dementias (ADRD) is scarce. PAFs refer to traits, actions, or assets that caregivers utilize to manage and conquer difficulties, pressures, or unfavorable situations in their caregiving role. Resilience is a common topic of interest, however there is less understanding of its significance in Black/American caregivers of individuals with ADRD. This qualitative study used narrative inquiry to explore cultural factors related to resilience among Black/African American ADRD partner caregivers. An adapted Resilience Framework informed this study and in-depth interviews with 10 Black/African American ADRD partner caregivers. Four major themes emerged: (1) Process of ADRD discovery, (2) Resilience in the caregiving process, (3) Challenges & facilitators to caregivers’ resilience, and (4) Race, ethnicity, cultural impact on caregivers’ resilience. The major themes include a total of 18 subthemes. Data from this study were compared to data from a previous pilot study that used similar questionnaire with a predominantly White sample of ADRD partner caregivers. Findings from this study contribute to our understanding of how race, ethnicity, and cultural factors influence resilience among diverse ADRD partner caregivers.

